# Removing barriers to management of adults with type 2 diabetes on insulin using continuous glucose monitoring in UK primary care practice: An expert consensus

**DOI:** 10.1111/dme.15500

**Published:** 2024-12-15

**Authors:** Samuel Seidu, Lorraine Avery, Heather Bell, Pam Brown, Jane Diggle, Su Down, Ritesh Dua, Patrick Holmes, Rahul Mohan, Nicola Milne, Thinzar Min, James Ridgeway, Waqas Tahir, Sanjay Tanna

**Affiliations:** ^1^ Diabetes Research Centre, National Institute for Health Research, Applied Research Collaboration East Midlands University of Leicester Leicester UK; ^2^ Solent NHS Trust Portsmouth UK; ^3^ Old School Surgery Carrickfergus UK; ^4^ Swansea London UK; ^5^ College Lane Surgery Ackworth UK; ^6^ Somerset Partnership NHSFT London UK; ^7^ Wye Valley NHS Trust Hereford UK; ^8^ St. George's Medical Practice Darlington UK; ^9^ Church House Surgery Ruddington UK; ^10^ Greater Manchester Diabetes Clinical Network Manchester UK; ^11^ Brooklands and Northenden Primary Care Network Wythenshawe UK; ^12^ Singleton Hospital and Neath Port Talbot Hospital Swansea Bay University Health Board Sketty UK; ^13^ University Hospitals Leicester NHS Trust Leicester UK; ^14^ Affinity Care Thornton & Denholme Medical Centre Bradford UK; ^15^ Blackpool Victoria Hospital Blackpool UK

**Keywords:** continuous blood glucose monitoring, primary care, type 2 diabetes

## Abstract

**Aims:**

This expert consensus reviews the reality of primary care clinical management of people with type 2 diabetes (T2D) on non‐intensive insulin therapy, with an emphasis on the use of continuous glucose monitoring (CGM) technology for effective care in this participant group. Here, we identify key unmet needs for skills and systems development within this frontline healthcare setting, along with major challenges and opportunities associated with managing these changes effectively.

**Methods:**

The authors participated in two primary care consensus panels held on 28 November 2023 and on 21 May 2024. The focus for these expert panels was to understand the unmet needs within primary care to manage adults with T2D treated with non‐intensive insulin therapy and incorporating the use of CGM systems. A Delphi Survey was undertaken among a wider group of Primary Care Diabetes Technology Network members in the United Kingdom, to understand prevalent attitudes to management of adults with T2D on insulin and using CGM in primary care. Based on these activities, a series of consensus statements were tested in a second Delphi Survey.

**Results:**

The activities described, involving primary care healthcare professionals (HCPs) with expertise in diabetes management, identified a series of training and educational needs within UK general practice that are central to skills development for the care of adults with T2D on insulin therapy and the application of CGM technology. Potential barriers to effective primary care management of people with T2D using CGM devices were identified. Areas of concern included confidence in national and local guidelines for the management of T2D using CGM systems, lack of experience on the part both of HCPs and people with T2D, clinical workflows and systems, as well as inbuilt resistance to change among primary care teams. However, the expert group were clear that the goal of providing care for people with T2D on non‐intensive insulin therapy using CGM technology as standard of care could be met (94.3%, *n* = 33). This will deliver clinical benefits for people with T2D, and improvements to clinical workflows in primary care. Cost‐savings to the health service were also identified as an outcome.

**Conclusions:**

The need to adapt to the management of people with T2D on insulin therapy puts significant pressure on current workflows and skills for primary care teams. Steps in overcoming these immediate pressures, to ensure effective clinical management of people with T2D, are discussed, along with a series of consensus statements that identify the key areas of change to manage. Ultimately, the great majority of expert primary care HCPs were confident or very confident that using CGM technology will become the standard of care for people with T2D treated with insulin in primary care.

**What's new?**

**What is already known?**

- Therapeutic inertia is an acknowledged factor in the failure of people with type 2 diabetes (T2D) on insulin and non‐insulin therapies to meet glycaemic targets.
- Managing people with T2D using CGM is challenging in the primary care setting.

**What this study has found**

- Primary care professionals with expertise in CGM identified barriers to its application in the management of people with T2D in UK general practice, including: lack of experience, confidence in guidelines, resistance to change.
- A Delphi Survey methodology was used to develop a series of consensus statements in this context.

**What are the implications of the study?**

- Education and training in the use of CGM in T2D are critical unmet needs for primary care teams in the United Kingdom.
- Primary care teams must be supported to use CGM technology in T2D, including the integration and use of CGM data as part of electronic health records.

## 1 INTRODUCTION

According to national diabetes audit data within the UK for 2024, more than 3.6 million people are living with type 2 diabetes (T2D).^1^, ^2^ National Institute for Health and Care Excellence (NICE) guidelines for the management of T2D in adults (NG28)^3^ recommend an HbA1c target of 53 mmol/mol (7.0%) or less for people on glucose‐lowering treatment and to intensify treatment if HbA1c rises to 58 mmol/mol (7.5%) or higher. Notably, NICE guidance also recommends measuring HbA1c every 3–6 months until HbA1c is stable and 6‐monthly reviews thereafter, with measurement of HbA1c at each review.^3^ National audit data show that approximately 50% of adults with T2D have an HbA1c >53 mmol/mol (>7.0%) and 36% have a last‐recorded HbA1c >58 mmol/mol (>7.5%).^1^ The national recommendations for use of continuous glucose monitoring (CGM) devices in T2D are also set by NICE, as we will discuss below.

One reason acknowledged for the low achievement of glycaemic targets is therapeutic inertia, defined as ‘the failure of healthcare professionals to intensify or deintensify therapy when appropriate to do so’.^4^ An important observation comes from a retrospective study of 2501 adults with T2D in the United Kingdom, with an HbA1c ≥64 mmol/mol (≥8.0%), which showed that 25% of the cohort did not have their treatment intensified to include insulin for at least 1.8 years, and 50% were not initiated on insulin therapy for almost 5 years after not achieving the target HbA1c range.^5^ This treatment inertia poses significant health risks for individuals with T2D. The United Kingdom Prospective Diabetes Study (UKPDS) Post Trial Monitoring Study^6^ showed that intensive control of HbA1c early after diagnosis of T2D creates a legacy effect that significantly reduces relative risks for myocardial infarction (by 17%) and death from any cause (by 10%), up to 24 years following the study end. Both the ADVANCE and the Glucose Control and Vascular Complications in Veterans with Type 2 Diabetes (VADT) studies have confirmed that intensive glycaemic control, with reduced HbA1c, is associated with significant reductions in microvascular and macrovascular complications of T2D, although the participants in these studies had long‐standing T2D.^7^, ^8^ Therapeutic inertia in T2DM also represents a significant economic burden in the United Kingdom. Using the IQVIA Core diabetes model, the increased costs of diabetes‐related complications and lost workplace productivity associated with treatment inertia, compared with achieving good glycaemic control (HbA1c 53 mmol/mol, 7.0%) over a 10‐year time horizon, corresponds to an additional economic burden of £2.6 billion.^9^

Therapeutic inertia in T2D is multifactorial and can be related to healthcare professional (HCP) and participant behaviours, as well as system‐related causes.^10^ However, a 2024 literature review of 22 studies^11^ concluded that a major driver of inertia for treatment intensification with insulin was fear of hypoglycaemia on the part of physicians, along with so‐called ‘psychological insulin resistance’ on the part of people with T2D related to their beliefs and perceptions of insulin and hypoglycaemia.^12^

The proportion of CGM use in people with T2D is relatively low compared to people with T1D but CGM uptake by people with T2D is increasing rapidly, with most growth in primary care, including among people with non‐insulin treated T2D.^13^, ^14^ The use of CGM systems can help manage concerns over hypoglycaemia, both for people with T2D treated with basal insulin and their primary care HCPs. Wearing a CGM sensor provides the person with T2D with biofeedback on their glucose levels in real time, along with clear information on whether their glucose levels are falling and how fast, using visible trend arrows. In discussion with their HCP, individuals with T2D can understand their daily proportion of time below range (TBR) with low glucose <3.9 mmol/L (<70 mg/dL) and how to minimise it.

There is significant evidence that using CGM in managing people with T2D on basal insulin or on non‐insulin therapy has benefits for glycaemia and beyond. These include reduced HbA1c,^15^, ^16^, ^17^ reduced total daily dose (TDD) of insulin,^18^, ^19^ weight loss,^19^ reduced hospital admissions for DKA or severe hypoglycaemia,^20^, ^21^ improvements in treatment satisfaction and self‐reported diabetes‐related behaviours.^19^ The 2021 MOBILE randomised controlled trial (RCT), recruited individuals with T2DM on basal insulin (*n* = 175), and showed that using CGM for an 8‐month period was associated with significantly reduced HbA1c, lower time above range (TAR) in hyperglycaemia >13.9 mmol/L (250 mg/dL) and reduced rates of hypoglycaemia events, compared with a control group using self‐monitored blood glucose (SMBG) testing alone.^17^ These data are consistent with results of retrospective studies demonstrating significant reductions in HbA1c for people with T2DM on basal insulin therapy.^15^, ^22^ The nationwide retrospective RELIEF study in France has shown that hospitalisations for acute diabetes events (ADEs) are reduced by −63% in the 12 months following initiation of CGM and by −70% in the 24 months following initiation, compared to the 12 months prior to initiation.^20^

The goal of this expert consensus is to review and discuss the reality of primary care clinical management of people with T2D on non‐intensive insulin therapy, with an emphasis on the wider use of CGM devices within the definitions provided in NICE NG28 for insulin management in this participant group. Since individuals with T2D on non‐intensive insulin therapy are likely to be managed in primary care, rather than in secondary care diabetes and endocrinology services, we also identify key unmet needs for skills and systems development in the primary care setting, in support of this goal, along with major challenges and opportunities associated with managing these changes effectively.

## 2 MEETING THE CHALLENGE OF MANAGING PEOPLE WITH T2D ON BASAL INSULIN OR PREMIXED INSULIN THERAPY IN PRIMARY CARE IN THE UK

The prevalence of T2D and its associated comorbidities is increasing. In the 5‐year period from 2019 to 2024, the number of people with a diagnosis of T2D in the United Kingdom increased by 9.8%.^23^, ^24^ The landscape of managing people with diabetes in the United Kingdom is also changing rapidly, with associated changes to the distribution of care for people with T2D. Endocrinology and diabetes services in the hospital outpatient setting are seeing the impact of introducing newer diabetes technologies for the care of children and adults with T1D on sensor‐augmented continuous subcutaneous insulin infusion (CSII) pumps and hybrid closed‐loop (HCL) automated insulin delivery (AID) devices. Primary care teams with experience of managing people with T2D on non‐insulin therapies face the challenge of taking over the clinical management of people with T2D on insulin therapy, and a position statement by Primary Care Diabetes Europe has set out a disease‐state model for what may be achieved by primary care teams through shared decision consultations with their patients.^25^ It is estimated that approximately 9% of people with T2D in the United Kingdom are on basal insulin or premixed insulin therapy.^26^ which would comprise around 325,000 individuals. A key goal is to be able to incorporate the care of this group of adults with T2D into UK primary care, without increased costs or burden of care. The application of CGM technology for individuals with T2D on non‐intensive insulin therapy has the potential to significantly enhance their care, and provide primary care teams with the tools to understand day‐to‐day glycaemic health status metrics in greater detail. However, this would mean using CGM‐derived metrics, such as time in range (TIR) 3.9–10.0 mmol/L (70–180 mg/dL), time above range (TAR) >10.0 mmol/L (>180 mg/dL), TAR >13.9 mmol/L (>250 mg/dL) and time below range (TBR) with low glucose <3.9 mmol/L (<70 mg/dL) and TBR <3.0 mmol/L (<54 mg/dL), in conjunction with a current HbA1c test result.

This consensus document examines the reality of primary care clinical management of people with T2D on basal insulin therapy, with an emphasis on the application of CGM technology for effective care in this participant group. We identify key unmet needs for skills and systems development within this frontline healthcare setting, along with the major challenges and benefits of succeeding in this endeavour. The consensus aims to bridge the identified gaps and provide actionable recommendations to enhance the clinical management of T2D people on insulin therapy using CGM technology in primary care settings.

## 3 METHODOLOGY

This consensus opinion reflects the outputs of a series of activities initiated within the Primary Care Diabetes Technology Network (DTN) in the United Kingdom. The named authors of the consensus opinion were each members of the primary care DTN, and self‐selected for participation in two primary care consensus panels held on 28 November 2023 and on 21 May 2024. The consensus group comprised seven primary care physicians (SS, HB, PB, RD, PH, RM, WT), five diabetes nurse practitioners (LA, JD, SD, NM, JR), one specialist clinical pharmacologist (ST) and one secondary care physician (TM). The focus for these expert panels was to understand the unmet needs within primary care to manage adults with type 2 diabetes (T2D) treated with insulin and incorporating the use of continuous glucose monitoring (CGM) systems. In conjunction, an initial Delphi Survey was undertaken among a wider group of primary care diabetes technology network members, to understand and interpret the prevalent attitudes to management of adults with T2D on insulin and using CGM in primary care. The Delphi process is a widely used,^27^, ^28^ validated technique for developing an expert consensus on clinical needs and approaches. Based on these activities, a second Delphi Survey was conducted, in which a series of consensus statements were offered for the members of the author group to agree or disagree with, based on a five‐point Likert scale. Consensus statements on which at least two‐thirds of the respondents agreed or strongly agreed with are presented in this paper in the discussion section.

The first Delphi Survey comprised an 18‐item questionnaire centred on the management of individuals with T2D treated with insulin in the primary care setting, and on the application of any CGM system. The survey used a mix of questions using a 5‐item Likert response format and also free‐text answers. Invited participants were all HCPs with demonstrated experience of managing individuals with diabetes and expertise in application of CGM systems and interpretation of CGM data. Responses were solicited that reflected the participants own experience and their attitudes to the opportunities and challenges faced by the wider primary care environment.

### 3.1 First Delphi Survey respondent characteristics

Overall, 35 individuals participated in the first Delphi Survey (Table S1). Respondents had a mean 14.7 years (SD 9.6 years) experience in primary care. In all, 32 respondents worked in primary care settings, and 3 practiced in a hospital setting. In this study, 48.6% of the respondents had experience of initiating CGM in at least 50 people with diabetes and 40.0% had experience with 20–50 CGM starts (Table S2).

## 4 MANAGING PEOPLE WITH T2D ON INSULIN IN PRIMARY CARE—OUTCOMES FROM A DELPHI SURVEY

Among the expert panel and the participants in the Delphi attitudes survey, the frequency of review with individuals with T2D on insulin therapy was consistent with the NICE guidelines for the management of T2D in adults, with 65.7% of respondents indicating that reviews were held at least every 6 months (Table S3). The remainder (34.3%) indicated a review was held at least every 12 months or when additional therapy was to be considered. Additionally, insulin users with T2D were assessed for hypoglycaemia risk on a regular basis, with at least 80% of survey participants reporting frequent assessments for recurrent hypoglycaemia (80.0%) or severe hypoglycaemia (88.6) (Table S4). The most common tools for assessing hypoglycaemia risk were person‐reported episodes (97.1%) and CGM‐generated ambulatory glucose profiles (88.6%) (Table S5). Emergency attendance records and fingerprick glucose meter readings were also important sources of information on hypoglycaemia risk (80.8% of respondents in both cases). Notably, expert primary care professionals reported assessing people with T2D on insulin for impaired awareness of hypoglycaemia (IAH), either frequently (65.4%) or sometimes (26.9%), typically using the Gold score (Table S5).

### 4.1 Using CGM for the management of people with T2D on insulin in primary care

An important discussion among the consensus panel was the interpretation of NICE guideline NG28 for the management of T2D in adults^3^ in regard to prescribing CGM sensors for adults with T2D not on intensive insulin therapy with multiple daily injections (MDI). The guideline recommends the use of intermittently‐scanned continuous glucose monitoring (isCGM) for adults with T2D on MDI, if any of the following apply: (a) they have recurrent hypoglycaemia or severe hypoglycaemia; (b) they have impaired awareness of hypoglycaemia (IAH); (c) they have a condition or disability that means they cannot self‐monitor their own capillary blood glucose; (d) they would otherwise be advised to self‐monitor their own capillary blood glucose at least eight times per day. Furthermore, NG28 recommends that adults with T2D should be offered isCGM if they are treated with any insulin therapy and would otherwise need help from a care worker or healthcare professional (HCP) to monitor their capillary blood glucose.

Significantly, the guideline does not define MDI as intensive basal‐bolus therapy, rather it clarifies that MDI indicates two or more daily insulin injections, which could be a basal‐bolus or basal plus regimen or twice‐daily mixed or other insulin. The Delphi Survey participants were asked to indicate their interpretation of this element of the guideline, as it refers to individuals in their own practices (Table S6). Responses made it clear that, among the people with T2D on insulin in their care, MDI would be interpreted to mean: basal‐bolus insulin therapy (74.3%), basal‐only insulin therapy requiring rescue injections of rapid‐acting insulin as appropriate (68.6%), basal‐only insulin therapy with the total daily dose split into two separate injections (77.1%) or use of premixed insulin given as two daily injections (80.0%). Significantly, the expert opinion of 97.1% of respondents was that CGM devices should be reimbursed for any person with T2D on any insulin therapy, and 34.3% indicated that CGM should be prescribed for any person with T2D at risk of hypoglycaemia on any therapy (Table S7), whether insulin or non‐insulin. This also highlights the regional inequity of guidance for use of isCGM in diabetes, since the Health Technology Wales guidance for use of isCGM in management of T1D or T2D mandates its use for all persons with diabetes on any insulin therapy, not just MDI, and does not include any qualifying statements regarding risks for hypoglycaemia.^29^

In a follow‐up question, Delphi Survey respondents were asked to provide feedback on the available CGM prescribing guidelines for people with T2D. The NICE NG28 guidelines were seen as well‐structured for use in primary care by 48.6% of respondents, with 28.0% disagreeing with this (Table S8). Guidelines provided by the local integrated care system (ICS) or health board were seen as providing clear guidelines for prescribing CGM in T2D by 40.0% of respondents, with 42.9% disagreeing with this assessment. Significantly, when asked whether primary care teams are concerned about being penalised for prescribing CGM for people with T2D outside of guidelines, 57.2% agreed that this was a concern, with only 17.2% indicating it was not a concern (Table S8).

### 4.2 Skills development among primary care teams

Among primary care practitioners with expertise in clinical care of people with T2D on insulin, a range of opinions were expressed in regard to training for all primary care teams who will be tasked with care of people with T2D on insulin. The Delphi Survey outcomes indicated that 42.9% of respondents felt skills development in management of T2D was among the higher priorities (ranked 1 or 2), whereas 54.3% believed it was the lowest priority (Table 1). This split may reflect the opinion among more‐expert practitioners that management of people with T2D is an established part of clinical care for many practices, but that targets for glycaemic control can be hard to meet. However, providing clinical care for individuals with T2D treated with insulin was seen as an important training need for primary care teams, with 71.4% of respondents identifying this aspect of care as a high priority (Table 1). Training in interpretation of CGM data in conjunction with HbA1c was given a reasonable priority for support (37.1%), whereas making treatment changes based on these insights was not given a high priority for education (Table 1). It is not clear whether the latter issue was seen as a low priority for education because, once identified using CGM profiles, the necessary treatment changes were then understood to follow.

**TABLE 1:** Training needs for primary care healthcare professionals who manage people with T2D in the primary care setting.

	Relative importance (1 = most important, 5 = least important)				
	1	2	3	4	5
Managing people with T2D	34.29% (12)	8.57% (3)	2.86% (1)	0.00%	54.29% (19)
Managing people with T2D on insulin	34.29% (12)	37.14% (13)	8.57% (3)	20.00% (7)	0.00%
Understanding the impact of using CGM in T2D	17.14% (6)	11.43% (4)	42.86% (15)	17.14% (6)	11.43% (4)
Interpretation of CGM glucose data in T2D alongside HbA1c	8.57% (3)	28.57% (10)	28.57% (10)	28.57% (10)	5.71% (2)
Making treatment changes in T2D based on CGM glucose data alongside HbA1c	8.57% (3)	11.43% (4)	17.14% (6)	34.29% (12)	28.57% (10)

*Note*: Data indicate proportion of respondents (*n*).

Abbreviations: CGM, continuous glucose monitoring; T2D, type 2 diabetes.

### 4.3 Application of CGM in primary care management of individuals with T2D on insulin

Given the Delphi Survey was conducted among HCPs with experience in using CGM to manage people with diabetes in primary care, there was a high level of confidence in the impact of using CGM for people with T2D on insulin (Table 2) and 94.3% of respondents agreed or strongly agreed that using CGM supports better decision making in this participant group and can reduce therapeutic inertia in meeting glycaemic goals (97.14%). From a clinical organisational perspective, the improved opportunities for remote monitoring of this population of people with T2D were seen as an important benefit of using CGM (94.3%), providing multiple avenues for objective engagement with people. There was also clear agreement (88.6%) that CGM technology provides an opportunity for increased use of the diabetes digital ecosystem to improve clinical workflows in primary care (Table 2), but this was balanced by caution among 65.7% of respondents that making changes to clinical workflows to incorporate CGM technology was a potential barrier (Table 3).

**TABLE 2:** Attitudes towards application of CGM in primary care management of people with T2D on insulin therapy.

	Strongly disagree	Disagree	Neither agree nor disagree	Agree	Strongly agree
Use of CGM in primary care supports better informed decision making for people with T2D on insulin	5.71% (2)	0.00%	0.00%	8.57% (3)	85.71% (30)
The potential for remote monitoring and population health management is an important benefit of CGM technology	5.71% (2)	0.00%	0.00%	20.00% (7)	74.29% (26)
Use of CGM in the care of people with T2D on insulin can reduce treatment inertia in achieving glycaemic goals	2.86% (1)	0.00%	0.00%	11.43% (4)	85.71% (30)
Use of CGM and the digital ecosystem in the care of people with T2D on insulin can improve clinical workflows in primary care	5.71% (2)	0.00%	5.71% (2)	34.29% (12)	54.29% (19)

*Note*: Data indicate proportion of respondents (*n*).

Abbreviations: CGM, continuous glucose monitoring; T2D, type 2 diabetes.

**TABLE 3:** Potential barriers for implementation of CGM in primary care management of people with T2D on insulin therapy.

	Relative importance (1 = Most important, 5 = Least important)				
	1	2	3	4	5
Lack of confidence in national guidance on use of CGM in T2D	31.43% (11)	17.14% (6)	45.71% (16)	0.00%	5.71% (2)
Lack of experience in using and interpreting CGM in T2D	62.86% (22)	20.00% (7)	11.43% (4)	0.00%	5.71% (2)
Lack of local guidelines on using CGM in T2D	31.43% (11)	17.14% (6)	20.00% (7)	25.71% (9)	5.71% (2)
Restricted access to CGM by formulary managers	37.14% (13)	17.14% (6)	28.57% (10)	11.43% (4)	5.71% (2)
Making changes to clinical workflows to incorporate CGM technology	25.71% (9)	40.00% (14)	17.14% (6)	8.57% (3)	8.57% (3)
Resistance to change among primary care teams	42.86% (15)	28.57% (10)	17.14% (6)	5.71% (2)	5.71% (2)
Providing education for people with T2D starting on CGM	20.00% (7)	45.71% (16)	31.43% (11)	2.86% (1)	0.00%
Lack of standardised platforms for using CGM in alignment with established practice systems	25.71% (9)	31.43% (11)	28.57% (10)	8.57% (3)	5.71% (2)

*Note*: Data indicate proportion of respondents (*n*).

Abbreviations: CGM, continuous glucose monitoring; T2D, type 2 diabetes.

### 4.4 Challenges to primary care management of individuals with T2D on insulin using CGM

Despite the confidence among the expert group that initiating CGM for people with T2D on non‐intensive insulin therapy would be beneficial for their care, there were several important barriers that were identified (Table 3). Significant among these was the potential for resistance to change within primary care teams, identified by 71.4% of survey participants. Lack of experience in using and interpreting CGM in T2D was a significant potential barrier (82.9%), although this can be resolved with training, as discussed above. Similarly, providing education for people with T2D starting on CGM is identified as a potential barrier (65.7%).

Another area of concern for implementation of CGM for people with T2D are the patient management systems available to primary care teams and whether they are fit‐for‐purpose. Lack of patient management systems compatible with incorporating and using CGM data was seen as a potential barrier to implementing CGM in primary care by 57.1% of respondents (Table 3) and this reduced confidence that effective management of this group was achievable (Table 4).

**TABLE 4:** Confidence in primary care systems to incorporate management of people with T2D on insulin therapy using CGM.

	Strongly disagree	Disagree	Neither agree nor disagree	Agree	Strongly agree
Systems and tools for initiating CGM with people with T2D on insulin in primary care are consistent and well standardised	2.86% (1)	54.29% (19)	20.00% (7)	11.43% (4)	11.43% (4)
Established primary care patient‐management systems are compatible with incorporating CGM data	8.57% (3)	25.71% (9)	28.57% (10)	25.71% (9)	11.43% (4)
Primary care teams will be able to adapt to management of people with T2D on insulin using CGM	5.71% (2)	11.43% (4)	11.43% (4)	37.14% (13)	34.29% (12)
Additional resources will be required to support the primary care workforce in managing T2D using CGM	0.00%	5.71% (2)	20.00% (7)	17.14% (6)	57.14% (20)
Effective use of CGM will reduce diabetes‐related healthcare costs for this group of people with T2D	0.00%	0.00%	5.71% (2)	20.00% (7)	74.29% (26)

*Note*: Data indicate proportion of respondents (*n*).

Abbreviations: CGM, continuous glucose monitoring; T2D, type 2 diabetes.

Lack of confidence in national or local guidelines on using CGM in T2D was an important potential barrier to primary care implementation for 48.6% of the expert group (Table 3), echoing the previously discussed ambivalence towards these elements of CGM application in T2D. Related to this, 54.3% of the Delphi Survey group felt that restrictions on access to CGM by formulary managers could impact implementation of CGM for people with T2D on insulin (Table 3).

These potential barriers to effective care of people with T2D on insulin therapy each indicate an objective concern that helps to frame the possible solutions and 74.3% of survey participants agreed that additional resources will be needed to support primary care teams managing individuals with T2D on insulin using CGM (Table 4). Overall, the expert HCPs participating in the Delphi Survey expressed considerable confidence that primary care teams will adapt to managing insulin‐treated people with T2D using CGM (71.4%) and that this will reduce diabetes‐related healthcare costs for this participant group (94.3%). Ultimately, 94.3% of expert primary care HCPs were confident or very confident that using CGM technology will become the standard of care for people with T2D treated with insulin in primary care (Table S9).

### 4.5 Understanding the patient experience of using CGM technology

Although 65.7% of the expert diabetes HCPs who participated in the Delphi Survey identified education for people with T2D starting on CGM as a potential barrier (Table 3), they also reported a more favourable experience (Table S10). When asked to reflect on the CGM initiation process, 65.7% of respondents indicated that more than 70% of individuals with T2D on insulin found CGM initiation straightforward and intuitive. This is an important insight from a group of primary care HCPs experienced with initiating CGM for people with T2D. Additionally, the reported experience of initiating and educating new CGM starters with T2D was best achieved in one‐to‐one sessions (71.4%) or group starts with 2–10 new users (22.8%) (Table S11). A small proportion of virtual starts were reported by 5.7% survey participants.

Although CGM initiation was perceived to be intuitive among the new user group, there was not the same engagement with telehealth, including virtual consultations and managing CGM as part of wider connected apps (Table S12). For 42.9% of Delphi survey participants, fewer than 20% of individuals with T2D were considered as engaged with telehealth. However, there clearly are technology adopters in the participant population, since 25.7% of respondents did indicate that more than 40% of the people with T2D in their practices were engaged with telehealth at some level. The most common reasons for limited use of telehealth were centred on poor literacy with computers and smartphones, fear of technology and preconceptions of complexity. The needs for education will clearly have to address these barriers.

## 5 IDENTIFYING PRIORITIES FOR CGM PRESCRIBING FOR PEOPLE WITH T2D ON INSULIN THERAPY IN PRIMARY CARE

The expert panel agreed that a key barrier to wider access to CGM for individuals with T2D on non‐intensive insulin therapy is the lack of a clear value‐proposition that distinguishes the small proportion of individuals on basal or premixed insulin from the very large population of people with T2D on any therapy. Currently, ICS stakeholders equate the cost of access to CGM for people with T2D on insulin therapy with the cost for the total population of people with T2D. This is an important barrier to overcome. In acknowledgement of the realistic need for budget control in delivery of primary care services, the expert consensus panel identified priorities for application of CGM for individuals with T2D on non‐intensive insulin therapy, to better define for ICSs the contained number of CGM prescriptions that may be required. Key in providing access to CGM for any individual is to set clear targets for glycaemic improvement within a defined period after initiation of CGM. Failure to achieve targets may indicate that CGM can be discontinued. Subgroups of people with T2D on insulin therapy for managed access to CGM may include:

- Individuals who have not met treatment goals no matter what interventions or intensifications have been tried, particularly those with very high HbA1c. This group has the poorest outcomes if restricted to SMBG and application of CGM may support clinically impactful behaviour change.
- Young persons with T2D for whom behavioural change may be more likely and for whom CGM can have more impact on their quality of life and mental health. CGM has the potential to give them agency over their own diabetes, with significant long‐term impact on health and wellbeing.
- Individuals with recurrent or severe hypoglycaemia, who are fearful of treatment intensification. This is a core initiation criteria identified in NICE NG28.^3^
- People with T2D and mild learning difficulties, who may need support to achieve glucose targets but who can become motivated by seeing their numbers on a daily basis. This criteria is also identified in NICE NG28.^3^

It is important to acknowledge that for all patient groups, there is bias and inequity in healthcare for areas of economic deprivation, which affects prescribing in intensively and non‐intensively treated T2D.^30^, ^31^ Managing this aspect of therapy in diabetes care is an important consideration.

## 6 MEETING THE NEED FOR EDUCATION FOR PRIMARY CARE PRESCRIBING OF CGM IN INSULIN‐TREATED TYPE 2 DIABETES

As indicated by the outcomes from the Delphi Survey, even with confident interpretation of NICE NG28, increased primary‐care prescribing of CGM in T2D must be accompanied by education of primary care teams on insulin initiation and management. Currently, only approximately 20% of practices are believed to be initiating insulin. This is a fundamental unmet need that ultimately impacts people with T2D and a critical rate‐limiting step in their therapy.

Educational initiatives must ensure that primary care teams receive complete education on the application AND interpretation of CGM devices and data. Otherwise there is a danger that people with T2D using insulin are initiated on CGM but that effective review and interpretation of the CGM data in their ambulatory glucose profile (AGP) will not happen, with continued therapeutic inertia. Thus, education must encompass what using CGM on a daily basis tells the person with T2D and their HCP about glycaemic patterns, such that all aspects of glucose control can be interpreted in the context of an individualised care plan.

It is also important to recognise that the experience of a person with T2D on insulin is very heterogeneous. The need for individualised care requires that this awareness is embedded within primary care teams as application of CGM in the care of people with T2D increases. This means understanding youth with T2D, people with learning disabilities, older and/frail individuals. For example, younger individuals with T2D have a more‐aggressive form of disease,^32^ with poorer outcomes, that have a disproportionate effect on workforce absenteeism and societal costs.

## 7 DISCUSSION

Primary‐care teams in various healthcare economies have responded to the need to implement CGM in the care of people with diabetes in their practices,^33^, ^34^ but challenges exist concerning education for primary care HCPs in the context of initiating CGM devices and interpreting CGM data.^35^ The initiative reported here is among the first to investigate the barriers to wider application of CGM in UK general practice, specifically for individuals with T2D on non‐intensive insulin therapy, and the opportunities provided by successfully implementation. In common with other published research, there is an important need for education within the wider UK general practice population, certainly concerning the application of CGM devices and CGM data in diabetes care, but also the fundamentals of good clinical care for people with T2D on insulin therapy and non‐insulin therapies. Significantly, the experience of our expert panel and the participants in the Delphi attitudes survey suggests that education centred on CGM is not a higher priority than good diabetes care in T2D, but that both are necessary.

A significant unmet need identified here is for clearer national guidance on the prescribing of CGM for people with T2D on non‐intensive insulin therapy. For example, NICE guideline NG28 states that CGM prescribing should be considered for a person with T2D on MDI therapy at risk of hypoglycaemia, which could be interpreted narrowly as intensive insulin therapy.^3^ However, the guideline does make a clarifying definition of MDI as indicating two or more daily insulin injections, which could either be an intensive basal‐bolus regimen or simply more than one daily insulin injection. As we report, for more‐expert diabetes practitioners, the NICE definition of MDI can be interpreted to include a person with T2D on any insulin therapy, including basal‐only insulin treatment split into two daily doses, basal insulin therapy requiring rescue injections with rapid‐acting insulin, and twice daily premixed insulin. In addition, there is a need for consistent local guidelines from ICS and health boards across the United Kingdom. The expert respondents in our Delphi Survey were split down the middle, with positive opinions about local guidelines being matched by those who felt that local guidelines lacked clarity. In this environment, there was genuine concern among primary care teams of being penalised for CGM prescribing outside of local or national guidelines, or of pharmacy managers imposing restrictions on access to CGM devices.

Given the wealth of data available from CGM sensors on which treatment optimisation can be based, a significant barrier to effective CGM implementation for people is the perception that primary care patient management systems are not well suited to the task of integrating CGM data metrics into electronic health records (EHRs), for example the incorporation of periodic %TIR, %TBR and %TAR benchmarking and goal‐setting. Currently, only a minority of healthcare organisations globally have successfully integrated CGM data directly into EHRs, which impedes the wider adoption of CGM technologies.^36^ Integration of CGM data in EHRs with adjunct data from fitness and sleep trackers, meal‐planning apps, connected insulin pens and other PROs, such as anxiety and depression, may further improve management of people with T2D in primary care.^37^ There is an unmet need for studies centred on education and empowerment of HCPs managing people with T2D in primary care, with the full benefit of integration of CGM data into participants' primary healthcare records.

In this context, there was a clear perception among the Delphi Survey respondents that a significant barrier to incorporating CGM technology into care of people with T2D on insulin is resistance to change among primary care teams, whether based on time constraints of lack of familiarity with technology in this context. This could reflect aspects of organisational change that can be perceived as disruptive, such as changes to clinical workflows and patient management systems, along with the need for specific education and training on new technologies. Many of these issues are infrastructure and organisational challenges, rather than diabetes‐specific issues to solve, but they speak to the need to ensure that primary care services in the United Kingdom are resourced and supported to meet the challenge.

Another important point is the role of CGM in reducing therapeutic inertia in the management of T2D. The expert panel highlighted that using CGM supports better decision‐making and can reduce therapeutic inertia, helping to meet glycaemic goals more effectively. This highlights the need for consensus targets for CGM metrics that are clear, understandable, and actionable to provide a strong framework for clinical decision making in primary care.

Delayed treatment intensification at the primary‐care level is linked to the increased incidence of microvascular and macrovascular disease in T2D,^38^, ^39^ which are accompanied by higher direct and indirect healthcare costs as a consequence.^9^, ^40^ The use of CGM is known to reduce the time to treatment intensification in a primary care setting for people with T2D^41^ but this highlights a need for consensus targets for CGM‐metrics to manage T2D that are clear, understandable and actionable, and that can provide a strong framework for objective clinical decision‐making in primary care. Although CGM‐based targets have been proposed for all people with diabetes,^42^ those specific to T2D have focused on people on intensive insulin therapy and evidence‐based CGM targets specifically for individuals with T2D on non‐intensive therapy are an unmet need. Currently, only a few healthcare organisations have successfully integrated CGM data directly into electronic health records (EHRs), which impedes the wider adoption of CGM technologies.^36^ Integration of CGM data in EHRs with adjunct data from fitness and sleep trackers, meal‐planning apps, connected insulin pens and other PROs, such as anxiety and depression, may further improve management of people with T2D in primary care.^37^ Future studies need to be conducted to include education and empowerment of HCPs managing people with T2D in primary care with integration of CGM data into patients’ primary healthcare records.

**Table:** 

**Consensus statements** Comprehensive training programs should be implemented for primary care teams that are focused on the use and interpretation of CGM data, alongside insulin initiation and management, to enhance primary care team capabilities and confidence. The treatment benefits and cost‐effectiveness of CGM for individuals with T2D on basal or premixed insulin must be differentiated from the broader T2D population to overcome budgetary barriers and ensure targeted use of CGM. Health services (and their providers) should be proactive in the development and deployment of patient management systems that effectively integrate CGM data into Electronic Health Records, allowing for seamless data utilisation and improved patient outcomes. Primary care teams must be enabled to utilise CGM technology to support timely treatment intensification and reduce therapeutic inertia by providing actionable insights into patients' glycaemic patterns, thereby improving glycaemic control and reducing diabetes‐related complications.

## 8 CONCLUSIONS

The great majority of expert primary care HCPs who contributed to the outcomes reported here were confident or very confident that using CGM technology will become the standard of care for people with T2D on non‐intensive insulin therapy in primary care. However, we acknowledge that the survey participants and the author group constitute part of an expert community, and thus introduce bias into the outcomes. That said, the expert author group has identified a number of important barriers to be overcome, such that primary care teams can effectively accept the challenge of managing this group of people with T2D and leveraging the value of CGM technology. A key unmet need in providing wider access to CGM for people with T2D on non‐intensive insulin therapy in primary care is education, both on the fundamentals of insulin initiation and long‐term management of insulin therapy, as well as on the application of CGM sensors and the interpretation of CGM data. Other barriers are related to organisational inertia that then translates into therapeutic inertia for people with diabetes. These can relate to the need to adapt workflows in primary care to accommodate the additional numbers of people with T2D, with the dual extra need for optimised insulin management along with integrated CGM data. Institutional resistance to organisational change is also perceived to be a barrier.

## AUTHOR CONTRIBUTIONS

The authors as a group participated in one or both of the consensus panels and in the Delphi Surveys. The group represents expertise in clinical management of T2D on insulin and in the application of diabetes technology. The authors did not receive fees or honoraria for work on the manuscript. In order to avoid delays, all feedback at all stages was curated and consolidated by a professional writer with experience in diabetes. All named authors meet the International Committee of Medical Journal Editors (ICMJE) criteria for authorship for this article, take responsibility for the integrity of the work, and have given their approval for this version to be published.

## CONFLICT OF INTEREST STATEMENT

SS reports personal fees from Amgen, AstraZeneca, Napp Pharmaceuticals, Eli Lilly, Merck Sharp & Dohme, Novartis, Novo Nordisk, Roche, Sanofi and Boehringer Ingelheim. Additionally, SS reports grants from AstraZeneca, Sanofi, Servier and Janssen. HB has received honoraria from Abbot, Roche, Dexcom, AstraZeneca, Lilly, Boehringer‐Ingelheim, Napp, Novo Nordisk, Daiichi Sankyo. PB has received payment for providing education materials and running education meetings for Abbott. JD has participated in Advisory Boards for Abbott, Bayer, Roche, AstraZeneca, Tetris, Menarini, Boehringer‐Ingelheim and Lilly. She has provided educational sessions and received funding from Abbott, Amarin, AstraZeneca, Bayer, Boehringer Ingelheim, Eli Lilly, Novo Nordisk, Roche, Sanofi, Takeda and Tetris. RD has received fees for preparing education materials and running education meetings for Abbott. SD has received fees for preparing education materials and running education meetings for Abbott. PH has received advisory or speaker fees from Abbott, and speaker fees from Dexcom. NM has received funding for providing educational sessions and for advisory boards activities from Boehringer‐Ingelheim, AstraZeneca, Eli Lilly, Novo Nordisk, Sanofi, Napp, Abbott, MyLan, Roche, Ascensia, Menarini and Bayer. NM declares payments for providing education or for attending advisory boards for Boehringer Ingelheim, AstraZeneca, Lilly, NovoNordisk, Sanofi, Napp, Abbott, MyLan, Roche, Ascensia, Menarini and Bayer. TM has received payments for speaking or advisory boards for Abbott, AstraZeneca, Boehringer‐Ingelheim and Napp. JR has received fees for preparing education materials and running education meetings for Abbott, Dexcom and Insulet. WT reports advisory funding, speaker or consultancy fees from: Abbott, Amarin, AstraZeneca, Bayer, Boehringer‐Ingelheim, Bristol Myers Squibb, Dawn, Dexcom, Eli Lilly, Medtec Europe, Novo Nordisk, NAPP Pharmaceuticals Ltd., MSD and Roche, INRStar, Medtronic, Oberoi Consulting, Pfizer, Roche, Sanofi‐Aventis, Servier.

## Supporting information


**Data S1.**.
