# Supplementary material for: Removing barriers to management of adults with type 2 diabetes on insulin using continuous glucose monitoring in UK primary care practice: An expert consensus
Source: Diabet Med. 2024 Dec 15;42(3):e15500. doi: 10.1111/dme.15500 (PMC11823331; doi:10.1111/dme.15500)
Supplement: Supplementary file 1 — Data S1.. [file DME-42-e15500-s001.pdf]

Removing barriers to management of adults with type 2 diabetes on insulin using continuous glucose monitoring in UK primary care practice: an expert consensus

Samuel Seidu, Lorraine Avery, Heather Bell, Pam Brown, Jane Diggle, Su Down, Ritesh Dua, Patrick Holmes, Rahul Mohan, Nicola Milne, Thinzar Min, James Ridgeway, Waqas Tahir, Sanjay Tanna

Supplementary materials

Supplementary Table 1. Delphi Survey respondent characteristics

Respondents (n)	35
Primary care, n (%)	32 (91.4)
Hospital diabetes/ endocrinology clinic, n (%)	3 (8.6)
Mean years in practice (SD)	14.67 (9.56)

SD, standard deviation

Supplementary Table 2.

Please provide an estimate of how many people with T2D on any therapy you have actively initiated on CGM in your practice (select one)

Responses	n	%
Fewer than 20 people	4	11.43%
20-50 people	14	40.00%
50-100 people	7	20.00%
More than 100 people	10	28.57%

Supplementary Table 3.

In your practice, what is the real-world frequency of a clinical review with a person with T2D on insulin therapy to discuss their glycaemic management?

Responses	n	%
Less than every 12 months	0	0
At least once every 12 months	10	28.57
At least once every 6 months	23	65.71
Other	2	5.71

Supplementary Table 4.

How frequently do you assess hypoglycaemia risk for people with T2D on insulin in your practice?

	Frequently % (n)	Sometimes % (n)	Rarely % (n)	Never % (n)
Recurrent hypoglycaemia	80.00% (28)	20.00% (7)	0.00%	0.00%
Severe hypoglycaemia	88.57% (31)	11.43% (4)	0.00%	0.00%
Impaired awareness of hypoglycaemia	62.86% (22)	28.57% (10)	8.57% (3)	0.00%

Supplementary Table 5.

When assessing hypoglycaemia risks, what tools do you use? (Select all that apply)

Responses	n	%
Person-reported episodes	34	97.14%
Emergency attendance or admission records	28	80.00%
Blood glucose test results	28	80.00%
Gold scores	15	42.86%
Clarke scores	3	8.57%
CGM reports	31	88.57%
Other	1	5.71%

Supplementary Table 6.

Please indicate in the list below, which of the treatment options you consider may be interpreted to qualify as 'multiple daily insulin injections (MDI)', given that in the glossary of terms in NICE NG28, MDI is defined as *'Two or more daily insulin injections, which could either be a basal-bolus regimen or more than one daily insulin injection'*. (select all that apply)

Responses	n	%
Basal-bolus insulin therapy	26	74.29%
Basal-only insulin therapy requiring rescue injections of rapid-acting insulin as appropriate	24	68.57%
Basal-only insulin therapy with the total daily dose split into two separate injections	27	77.14%
Use of premixed insulin given as two daily injections	28	80.00%
Other	5	14.29%

Supplementary Table 7.

NICE NG28 indicates use of CGM for persons with T2D on MDI and either recurrent hypoglycaemia, severe hypoglycaemia or impaired awareness of hypoglycaemia. Which of the following statements reflects your expert opinion. (select all that apply)

Responses	n	%
CGM should be prescribed for people with T2D at risk of hypoglycaemia on basal-bolus insulin therapy	18	51.43%
CGM should be prescribed for people with T2D at risk of hypoglycaemia on any insulin therapy	34	97.14%
CGM should be prescribed for people with T2D at risk of hypoglycaemia on any therapy	12	34.29%

Supplementary Table 8.

Please indicate your level of agreement with the following statements

	**Strongly disagree % (n)**	**Disagree % (n)**	**Neither agree nor disagree % (n)**	**Agree % (n)**	**Strongly Agree % (n)**
The guidance for prescribing CGM in NG28 is well structured for use in primary care	2.86% (1)	25.17% (9)	22.86% (8)	34.29% (12)	14.29% (5)
My local ICS/health board/trust provides clear guidelines for prescribing CGM in T2D for primary care	20.00% (7)	22.86% (8)	20.00% (7)	20.00% (7)	17.14% (6)
Primary care teams are concerned about being penalised for prescribing CGM outside of guidelines in NG28	2.86% (1)	14.29% (5)	34.29% (12)	22.86% (8)	25.71% (9)

CGM, continuous glucose monitoring; T2D, type 2 diabetes

Supplementary Table 9.

Please indicate your level of confidence with the following statements as they relate to primary care within the next 3 years (i.e. by summer 2027)

	Not at all confident % (n)	Limited confidence % (n)	Confident % (n)	Very confident % (n)
CGM will be the standard of care for people with T2D on insulin therapy	00.00%	5.71% (2)	20.00% (7)	74.29% (26)
Effective care of people with T2D on insulin therapy in primary care will be routine	00.00%	22.86% (8)	34.29% (12)	42.86% (15)

Supplementary Table 10.

What proportion of new CGM users with T2D on insulin find the initiation process straightforward and intuitive?

Responses	n	%
None	0	0.00%
Less than 10%	0	0.00%
10-20%	3	8.57%
21-50%	6	17.14%
51-70%	3	8.57%
More than 70%	23	65.71%

Supplementary Table 11.

Based on your own experience, please indicate what you perceive to be the most effective format for initiating CGM in people with T2D on insulin therapy in your practice

Responses	n	%
One-to-one training and education	25	71.43%
Group starts with 2-10 individuals	8	22.86%
Group starts with more than 10 individuals	0	0.00%
Virtual starts	1	2.86%
Other	1	2.86%

Supplementary Table 12.

In your clinic, what proportion of persons with T2D on insulin therapy engage with telehealth services (diabetes apps, Zoom clinical appointments, CGM tools, etc.)

Responses	n	%
More than 50%	3	8.57%
40-50%	6	17.14%
30-40%	5	14.29%
20-30%	6	17.14%
Less than 20%	15	42.86%
